# Supplementary material for: Safety climate in the operating room in the pre-pandemic and pandemic period of COVID-19: A mixed method study
Source: PLoS One. 2024 Jul 19;19(7):e0305281. doi: 10.1371/journal.pone.0305281 (PMC11259288; doi:10.1371/journal.pone.0305281)
Supplement: Attachment — Submitted filename: Rebuttal letter.docx [file pone.0305281.s001.docx]

Rio de Janeiro, March 17, 2024

Dear  Academic Editor,

We would like to thank you for your careful review of the material submitted, which helped to improve the submission PONE-D-23-43150 “Safety climate in the operating room in the pre-pandemic and pandemic period of COVID-19: A mixed method study”.

The adjustments made are listed below. In addition, justifications were given for the points that have not been met or have been partially met.

Journal requirements:

When submitting your revision, we need you to address these additional requirements.

1. Please ensure that your manuscript meets PLOS ONE's style requirements, including those for file naming. The PLOS ONE style templates can be found at: **Adjustments made according to submission guidelines.**

2. When completing the data availability statement of the submission form, you indicated that you will make your data available on acceptance. We strongly recommend all authors decide on a data sharing plan before acceptance, as the process can be lengthy and hold up publication timelines. Please note that, though access restrictions are acceptable now, your entire data will need to be made freely accessible if your manuscript is accepted for publication. This policy applies to all data except where public deposition would breach compliance with the protocol approved by your research ethics board. If you are unable to adhere to our open data policy, please kindly revise your statement to explain your reasoning and we will seek the editor's input on an exemption. Please be assured that, once you have provided your new statement, the assessment of your exemption will not hold up the peer review process: **The data will be made available.**

| **Opinion issued** | **Attended** | **Amendments/Justifications** |
| --- | --- | --- |
| **ABSTRACT** | | |
| 1. Consider rephrasing the purpose of the study for clarity, such as specifying the focus on safety climate. | Yes | The objective of the study was reformulated to focus on the safety climate in the operating room. |
| 2. The convergence strategy is mentioned but not elaborated. Include a brief explanation of how the mixed-method approach was applied. | Yes | The methodological details are in the methods section following the submission guidelines |
| 3. Specify the duration of the pre-pandemic and pandemic periods. | Yes | Adjustments made. The pre-pandemic period of COVID was defined as March 2020, since the World Health Organization on March 11^th^, 2020, declared the novel coronavirus (COVID-19) outbreak a global pandemic. As for the pandemic period, this research considered the 2^nd^ and 3^rd^ global waves. |
| 4. Mention the institutional review board approval for ethical considerations. | Yes | Request granted. |
| 5. Provide more context regarding the Pillar Integration Process (PIP) for better understanding. | Yes | The methodological details are in the methods section following the submission guidelines |
| **Introduction** | | |
| 1. Clarify the relevance of analyzing safety climate in the context of COVID-19. | Yes | The discussion on safety climate in the context of COVID-19 becomes imperative considering that the pandemic has gradually impacted the surgical practice environment and the health organizations’ routines. This information is detailed in the introduction. |
| 2. Elaborate on the impact of COVID-19 on the safety climate. | Yes | In the introduction, we present the consequences of the COVID-19 pandemic in the operating room and the importance of reflecting on its impact on the safety climate. |
| 3. Improve flow and coherence in the introduction section for better readability. | Yes | Adjustments made |
| **AIM** | | |
| 1. The research problem is stated, but it could be more succinct. Consider refining the problem statement. | Yes | Adjustments made |
| 2. Clarify the Pillar Integration Process and its role in achieving the objectives. | Yes | The objectives have been reworded for clarity. |
| 3. Consider rephrasing objectives for better clarity and alignment with the study's focus. | Yes | The objectives were reformulated in line with the focus of the study: to verify whether the COVID-19 pandemic had an impact on the safety climate from the perception of the multiprofessional team in the operating room and to analyze the domains of the safety climate in the pre-pandemic and pandemic period of COVID-19 from the perception of the multiprofessional team in the operating room, highlighting the intersections of quantitative and qualitative approaches. |
| **Methods** | | |
| 1. Provide a concise rationale for choosing mixed-method research in the context of public health emergencies. | Yes | Given the complexity of the safety climate study, and the fact that the operating room is so affected by an atypical context such as the COVID-19 pandemic, the integration of quantitative and qualitative data permits analysis and conclusions to provide a complete view of the object under study, since the complexity of the research problem requires answers that go beyond statistical analysis or researching the meaning of a phenomenon. This information is detailed in the methods. |
| 2. Specify the role of the Pillar Integration Process in data analysis. | Yes | Request granted. The integration of quantitative and qualitative data permits analysis and conclusions to provide a complete view of the object under study. The process of carrying out data integration has been specified in the method. |
| 3. Clarify why the specific reporting guidelines and tools (STROBE®, COREQ, MMAT) were chosen. | Yes | To conduct the study and ensure greater reliability, we used the EQUATOR network tools, guaranteeing methodological rigor, quality, and transparency in scientific writing. We applied the STROBE tool for the cross-sectional quantitative phase, the COREQ for the qualitative phase and the MMAT for the integration of quantitative and qualitative approaches. |
| 4. Highlight the importance of intentional sampling in the qualitative phase. | Yes | Request granted |
| 5. Consider restructuring the section for improved organization and clarity. | Yes | Section restructured for better organization and clarity |
| **Overall** | | |
| 1. Ensure consistency in terminology and formatting throughout the document. | Yes | Request granted |
| 2. Consider adding more details about the characteristics of the study setting for better context. | Yes | More details of the study scenario have been added for better contextualization |
| 3. Address potential redundancies and streamline sentences for better readability. | Yes | Request granted |
| 4. Double-check the order and sequence of the content for logical flow. | Yes | Request granted |
| **Results** | | |
| **Quantitative Data** | | |
| 1. Clarify the rationale for choosing the 'Communication in the surgical environment' domain for discussion in the quantitative phase. | Yes | Request grantes. This result is relevant, as this domain is the differentiating factor of the SAQ/OR to SAQ. |
| 2. Ensure that the statistically significant associations in the 'Perception of stress' and 'Working conditions' domains are well-explained and discussed. | Yes | The statistically significant associations were better explained and discussed |
| 3. Clearly present the key findings and their implications for the safety climate during the pre-pandemic and pandemic periods. | Yes | Request granted. We present more clearly the main results and their implications for the security climate during the pre-pandemic and pandemic periods. |
| **Qualitative Data** | | |
| 1. Consider adding a brief summary or introduction to the qualitative findings to provide context before presenting the categories. | Yes | Request granted |
| 2. Provide a concise overview of the six categories and their respective proportions to facilitate readers' understanding and Discuss the significance of each category, emphasizing their impact on the safety climate in the operating room. | Yes | Request granted |
| **Data Integration** | | |
| 1. Clarify how the Pillar Integration Process (PIP) was employed for data integration, emphasizing its role in synthesizing the quantitative and qualitative findings. | Yes | Request granted. The PIP was detailed to clarify how the quantitative and qualitative data were synthesized and integrated. |
| 2. Clearly articulate the benefits and insights gained from integrating both types of data, emphasizing any convergences or divergences observed. | Yes | Request granted.This integration technique approach made it possible to comparatively assess the impact of COVID-19 on the safety climate in the operating room. |
| **Discussion** | | |
| 1. Begin the discussion by briefly reintroducing the six identified pillars or categories to provide a clear framework for the discussion. | Yes | Request granted. |
| 2. Further elaborate on the significance of nursing as the predominant category, especially in the context of the Brazilian healthcare system. Discuss how this dominance might influence the safety climate. | Yes | Request granted. We explore the importance of nursing in the context of the Brazilian healthcare system and its role as a driver of the safety climate. We describe the challenges posed by COVID-19 in the operating room environment. |
| 3. Explore in more detail the specific challenges faced by healthcare professionals during the COVID-19 pandemic. Discuss the implications of the pandemic on resources, staff management, and overall patient care. | Yes | We explore the challenges faced by the organization and healthcare professionals during the COVID-19 pandemic, and their impact on management and care. |
| 4. Provide a more in-depth discussion on the factors contributing to the negative perception in the 'Working Conditions' domain during the pandemic. Explore potential strategies to improve working conditions for healthcare professionals. | Yes | Request granted. |
| 5. Discuss the dichotomy between quantitative and qualitative findings regarding stress perception. Explore the reasons for this disparity and its implications for healthcare professionals' well-being and patient safety. | Yes | Request granted. The possible reason for the dichotomy of findings was clarified, we explored the importance of professional safety and its reflection on patient safety, and the influence of COVID-19 on the dynamics of the operating room. |
| 6. Explain the qualitative findings related to communication, addressing the identified need for improvement. Propose potential interventions or strategies to enhance communication within the surgical team. | Yes | Request granted. The requirements were met and improvement strategies for the identified weaknesses were proposed as a contribution to practice. |
| 7. Discuss the negative scores in the 'Safety Climate' domain and how these align with the qualitative findings in 'Safety Culture Maturity.' Propose recommendations for strengthening the safety culture in the operating room. | Yes | The requirements were met and improvement strategies for the identified weaknesses were proposed as a contribution to practice. |
| 8. Provide a nuanced discussion on the varying perceptions of management. Explore how management actions during the pandemic influenced safety culture and propose strategies for improving managerial support. | Yes | The requirements were met and improvement strategies for the identified weaknesses were proposed as a contribution to practice. |
| 9. Elaborate on the converging results regarding the fragility of the 'Fair Organizational Culture' pillar. Discuss the importance of a fair culture in promoting patient safety and suggest interventions for improvement. | Yes | A fair and mature safety culture is reflected in the actions that professionals will employ when working as a form of barrier to the occurrence of incidents resulting from care. We discussed the importance of this culture in the organization as a way to promote surgical patient safety. In addition, we suggested strategies for improvement. |
| **Limitations** | | |
| 1. Emphasize the limitations of the study, such as its single-center focus and potential memory bias. Discuss how these limitations may impact the generalizability of the findings. | Yes | Request granted. |
| **Conclusion** | | |
| 1.Summarize the key findings concisely and restate their implications for practice, management, and society. Highlight the study's contribution to understanding safety climate during the pandemic and suggest avenues for future research. | Yes | Request granted. |

We declare that the group of authors agrees with the changes made.

We remain at your disposal for any further clarifications or adjustments that may be necessary.

Kind regards,

Rosilene Ferreira
