# Supplementary material for: Safety climate in the operating room in the pre-pandemic and pandemic period of COVID-19: A mixed method study
Source: PLoS One. 2024 Jul 19;19(7):e0305281. doi: 10.1371/journal.pone.0305281 (PMC11259288; doi:10.1371/journal.pone.0305281)
Supplement: Attachment — Submitted filename: No comments.docx [file pone.0305281.s002.docx]

No comments
